# Surgical Diseases and Injuries of the Genito-Urinary Organs

**Published:** 1916-07

**Authors:** 


**Surgical Diseases and Injuries of the Genito-Urinary Organs.** By J. W. THOMSON WALKER, M.B., C.M. Edin., F.R.C.S. Eng. Pp. xviii., 879. London : Cassell & Company Ltd. 1914. Price 25s. net.

The diseases and injuries of the genito-urinary organs are here described in a thorough and lucid manner by one who is obviously a master of the subject. The author's personal experiences in dealing with the treatment of these diseases, which has been large, is freely drawn upon, and the opinions expressed and advice given bear the impress of being solidly based upon carefully-considered facts. The illustrations are excellent and numerous, and the coloured drawings of cystoscopic appearances are specially beautiful and illuminating. The book is likely to be the standard one on genito-urinary diseases in this country for many years to come.

